# Carbetocin versus oxytocin in the management of the third stage of labor for preventing postpartum hemorrhage after vaginal delivery

**DOI:** 10.1590/1806-9282.20241496

**Published:** 2025-06-16

**Authors:** Mustafa Goksu, Ozan Karadeniz

**Affiliations:** 1 Health Sciences University, Kanuni Sultan Suleyman Training and Research Hospital, Department of Obstetrics and Gynecology – İstanbul, Turkey.; 2 Başakşehir Cam and Sakura City Hospital, Department of Obstetrics and Gynecology – İstanbul, Turkey.

**Keywords:** Carbetocin, Oxytocin, Postpartum hemorrhage, Delivery, Obstetric

## Abstract

**OBJECTIVE:**

The aim of this study was to compare the effect of carbetocin and oxytocin administration on the prevention of postpartum hemorrhage concerning the estimated amount of bleeding, the need for additional uterotonics, and alterations in hemoglobin levels in patients undergoing vaginal delivery.

**METHODS:**

A cohort investigation was carried out at a tertiary hospital from November 2020 to December 2023, comprising women who underwent vaginal delivery and exhibited at least one risk factor for atonic postpartum hemorrhage. The study included pregnant women at gestational ages between 37 and 40 weeks. Following vaginal delivery, the participants were randomly assigned to receive either five units of oxytocin or 100 μg of carbetocin intravenously. Postpartum blood loss was subsequently measured objectively in milliliters using a calibrated bag attached to a postpartum drape.

**RESULTS:**

The peripartum estimated bleeding volumes for the oxytocin and carbetocin groups were 461.41±65.41 cc and 446.35±68.18 cc, respectively (p=0.096). There was no statistically significant difference observed between the two groups in terms of hemoglobin decrease and additional uterotonic requirement (p=0.904 and p=0.348, respectively).

**CONCLUSION:**

The findings suggest that carbetocin was equally effective and safe compared to oxytocin in preventing postpartum hemorrhage among women undergoing vaginal delivery.

## INTRODUCTION

Postpartum hemorrhage (PPH) presents a potentially life-threatening complication in both vaginal and cesarean deliveries. It is defined as a postpartum blood loss of 500 mL or more after vaginal delivery and a blood loss exceeding 1,000 mL after cesarean delivery^1^. It holds particular significance in developing nations, contributing significantly to maternal morbidity and mortality, constituting a quarter of global maternal deaths^2^. Roughly, 6% of all deliveries encounter PPH^3^. Despite notable advancements in its management, PPH continues to stand as a primary cause of maternal mortality worldwide.

Among other risk factors, uterine atony stands out as a major contributor to PPH^4^. Effective management of the third stage of labor plays a crucial role in preventing PPH. Uterotonic agents were used as a preventive strategy to reduce the occurrence of PPH^5^. Oxytocin, the most commonly used uterotonic agent for PPH prevention, is preferred and is effective in preventing PPH after both cesarean and vaginal deliveries. Continuous intravenous (IV) infusion is recommended for routine postpartum care due to its short half-life (<10 min) and common side effects, including decreased blood pressure, nausea, vomiting, cardiac arrhythmia, pulmonary edema, water intoxication, and seizure^6,7^.

Carbetocin (1-deamino-1-carba-2-tyrosine [O-methyl]-oxytocin) is the synthetic analog of an oxytocin agonist with a prolonged half-life of 40 min^8^. It has a rapid onset of action (2 min) and a long duration of action after administration^9^. A 100-μg dose of carbetocin is generally considered equivalent to five units of oxytocin^9,10^. Although there are some potential side effects, no serious adverse events have been reported for carbetocin. The unique profile and rapid action of carbetocin make it more advantageous in the management of the third stage of labor^4^. There is a lack of consensus regarding the superiority of one drug over the other. Therefore, we aimed to compare the effectiveness of IV carbetocin with that of IV oxytocin for the prevention of atonic PPH after vaginal delivery.

## METHODS

This study was conducted at the Department of Obstetrics and Gynecology of the Kanuni Sultan Suleyman Training and Research Center in Istanbul on 220 pregnant women aged 37–40 weeks who had vaginal deliveries between November 2020 and December 2023. Our study was approved by the Ethics Committee of the Health Sciences University, Istanbul Kanuni Sultan Suleyman Training and Research Hospital (approval number KAEK/2023.12.201). The study followed the ethics standards recommended by the Declaration of Helsinki. All participants were counseled after admission regarding the study protocol and objectives, after which written informed consent was obtained authorizing their participation in the study. Informed consent was obtained from all individual participants included in the study.

An electronic medical database of the hospital was utilized to identify patients who presented to our labor ward between 37 and 40 gestational weeks upon the onset of labor and subsequently underwent vaginal delivery. The exclusion criteria were as follows: patients having placenta previa/accreta, placenta abruption, myoma uteri, twin gestation, coagulopathy, cardiac disease, and liver and kidney diseases; patients using anticoagulants or aspirin; patients having diabetes or gestational diabetes mellitus; and patients with pregnancy hypertension. The patient's preoperative evaluation comprised demographic characteristics (age, body mass index [BMI], gravida, and parity), pre-labor hemoglobin levels, and birth weight. Postpartum evaluation included additional uterotonic agent usage, post-delivery hemoglobin levels, estimated blood loss, and hemoglobin decrement exceeding 10%. In view of the impact of socioeconomic characteristics such as lifestyle and dietary habits on anemia and bleeding diathesis, we also compared the socioeconomic status of the groups.

In all cases, placental cord traction and uterine massage were performed to deliver the placenta. Two separate groups of patients were designated for preventing PPH: the first group received carbetocin (Pabal®, Ferring, Germany), while the second group was given oxytocin (Synpitan Forte®, Deva Pharmaceuticals, Turkey). Patients in the oxytocin group received a total of 20 IU IV through a slow injection over 1 min immediately after placental delivery. On the other hand, patients in the carbetocin group received a single IV dose of 100 μg immediately following placental delivery. Postpartum blood loss was subsequently measured objectively in milliliters using a calibrated bag attached to a postpartum drape.

The patients were informed regarding the cost and benefits of each drug (oxytocin or carbetocin) for prevention, and the decision regarding medication choice was left to them. The drugs were administered to the patients following placental delivery. Additional uterotonics were administered to patients exhibiting insufficient uterine tonus during the postpartum period. The administration of additional uterotonics within 24 h postpartum (ergot alkaloids, misoprostol, etc.) was documented. Patients were assessed based on estimated blood loss, the requirement for additional oxytocin, and hemoglobin levels.

### Statistical analysis

For the analysis of research data, SPSS Inc., Chicago, USA (SPSS v28.0) software was used. Descriptive statistics for categorical variables in the study were provided as frequency and percentage. In contrast, descriptive statistics for quantitative variables were given as mean and standard deviation: Pearson's χ^2^ test and Fisher's exact test examined relationships between categorical variables. The data that follow a normal distribution were compared with the Student's t-test, while Mann-Whitney U test was used for the data that did not follow a normal distribution. The standard distribution suitability of quantitative variables was investigated using the Kolmogorov-Smirnov test. A statistical significance threshold of p<0.05 was adhered to throughout the analysis.

## RESULTS

A total of 220 patients who delivered vaginally in our delivery ward were included in our study. While 110 of these patients were administered oxytocin, 110 of them were applied carbetocin. There was no statistically significant difference among groups in terms of demographic and clinical characteristics (age, BMI, gravida, parity, pre-labor hemoglobin levels, and birth weights of newborns) (p>0.05) (Table 1). No statistical significant difference was observed between the two groups in terms of socioeconomic status (p=0.548) (Table 2).

**Table 1:** Demographic and clinical characteristics of the patients.

	Group 1: Oxytocin group (n=110)	Group 2: Carbetocin group (n=110)	p-value
Age (year)	28.49±1.95	28.41±2.08	0.755
BMI (kg/m^2^)	28.27±3.90	28.72±2.73	0.321
Gravida	1.92±0.92	1.85±0.91	0.606
Parity	0.86±0.89	0.83±0.90	0.763
Nulliparous	49 (44.5%)	52 (47.3%)	0.685
Multiparous	61 (55.5%)	58 (52.7%)	
Antenatal Hb value (g/dL)	11.85±0.97	11.68±0.76	0.167
Newborn weight (g)	3,000±191	2,991±280	0.415

Data are mean±standard deviation or n (%); Hb: hemoglobin; BMI: body mass index.

**Table 2:** Comparison of socioeconomic status between groups.

	Group 1: Oxytocin group (n=110)	Group 2: Carbetocin group (n=110)	p-value
Low	48 (43.6%)	54 (49.1%)	0.548
Middle	52 (47.3%)	44 (40.0%)	
High	10 (9.1%)	12 (10.9%)	

Data are expressed as number (%).

The peripartum estimated bleeding volumes for the oxytocin and carbetocin groups were 461.41±65.41 cc and 446.35±68.18 cc, respectively (p=0.096) (Table 3). There was no statistically significant difference observed between the two groups in terms of hemoglobin decrease and additional uterotonic requirement (p=0.904 and p=0.348, respectively). Notably, 55.5% of the patients in the oxytocin group and 58.2% of the patients in the carbetocin group experienced a postpartum decrease in hemoglobin levels exceeding 10% (p=0.683).

**Table 3:** Comparison of postpartum outcomes between the two groups.

	Group 1: Oxytocin group (n=110)	Group 2: Carbetocin group (n=110)	p-value
Additional uterotonic requirement	8 (7.3%)	12 (10.9%)	0.348
Decrease in Hb (g/dL)	1.20±0.35	1.19±0.28	0.904
Number of patients with more than 10% Hb decrease	61 (55.5%)	64 (58.2%)	0.683
Estimated amount of bleeding (mL)	461.41±65.41	446.35±68.18	0.096

Data are mean±standard deviation or n (%), Hb: hemoglobin.

## DISCUSSION

The main objective of our study was to determine whether oxytocin or carbetocin was associated with a decrease in postpartum blood loss at the time of vaginal delivery. The results of our study suggest that neither carbetocin nor oxytocin demonstrated superiority over the other in preventing PPH after vaginal delivery. Additionally, no significant difference was observed between the groups in terms of additional uterotonic usage, postpartum hemoglobin decline, or estimated blood loss.

There is limited research on the utilization of carbetocin following vaginal delivery, and the available data are controversial. Several studies have highlighted carbetocin's potential superiority over IV oxytocin in preventing PPH^11,12^. Conversely, Vernekar et al. suggested that heat-stable intramuscular (IM) carbetocin may lead to slightly lower postpartum hemoglobin levels and a higher drop in hemoglobin levels (>2 g/dL)^13^. Moreover, Liu et al., in their study comparing IV carbetocin to oxytocin after vaginal delivery in high-risk pregnancies, discovered no significant difference in terms of postpartum bleeding (>500 mL) and the volume^14^. Amornpetchakul et al. conducted a triple-blinded randomized controlled trial comparing IV carbetocin and IV oxytocin following vaginal delivery in high-risk singleton pregnancies. They concluded that carbetocin is more effective in preventing PPH in singleton pregnancies with at least one risk factor for PPH^11^. Maged et al. reported that IV carbetocin 100 mg is a safe option for managing atonic PPH, with non-significant hemodynamic changes or side effects compared to IV oxytocin^12^. In another randomized controlled trial by Cetin et al., carbetocin emerged as a superior option to oxytocin and oxytocin plus tranexamic acid regimens for PPH prophylaxis after vaginal delivery^15^. The conflicting results among oxytocin and carbetocin studies may be attributed to variations in oxytocin dosage and differences in patient selection. According to our study, we found out that IV carbetocin is not favorable against IV oxytocin for preventing PPH after vaginal delivery. The observed outcome can be attributed to our utilization of a 20-unit slow infusion of oxytocin for PPH prophylaxis, whereas most studies employed a 5-unit IV oxytocin regimen, which might have been inadequate for effective prevention. In parallel with our study, the CHAMPION trial conducted by Widmer et al. involving 29,645 women who underwent vaginal deliveries revealed that carbetocin was non-inferior to oxytocin concerning blood loss (>500 mL) or the requirement for additional uterotonic agents for PPH prevention^16^. Furthermore, a meta-analysis involving 30,134 women comparing carbetocin versus oxytocin for the prevention of PPH after vaginal delivery demonstrated that carbetocin was equally effective and safe as oxytocin in preventing PPH during vaginal delivery^16^. Indeed, the collective findings suggest that the selection of a uterotonic agent for PPH prevention during vaginal delivery should be based on a cost-effective assessment and individualized for each case.

The interpretation of this study is subject to some limitations. The weaknesses of our study are that a limited number of patients were examined and the side effects of the drugs used were not reported. The strengths of our study are that only patients with vaginal deliveries were examined, deliveries took place in a single center under the supervision of the same team, and the clinical and socioeconomic characteristics of the two groups were statistically similar. It is obvious that our knowledge about these two drugs will be strengthened with more comprehensive studies in the future.

## CONCLUSION

Carbetocin demonstrated comparable effectiveness and safety to oxytocin in preventing PPH among women undergoing vaginal delivery. The decision to use carbetocin as an uterotonic for routine prophylaxis will depend on its cost-effectiveness.

## Data Availability

All data relevant to this study will be provided by the authors upon specific request.
